# Impact of Co-Inoculation Patterns of *Wickerhamomyces anomalus* and *Saccharomyces cerevisiae* on Cider Quality and Aromatic Profiles

**DOI:** 10.3390/molecules30071620

**Published:** 2025-04-04

**Authors:** Yue Wei, Jianguo Mou, Haoran Zhang, Aiying Gao, Yi Qin

**Affiliations:** 1 College of Enology, Northwest A&F University, Yangling 712100, China; wy031619@163.com (Y.W.); zywmsjb@163.com (J.M.); wanguo1103@163.com (H.Z.); 2 Taian Institute for Food and Drug Control (Taian Fiber Inspection Institute), Taian 271000, China; aygao@126.com; 3 National Forestry and Grassland Administration Engineering Research Center for Viti-Viniculture, Yangling 712100, China; 4 Heyang Viti-Viniculture Station, Northwest A&F University, Heyang 715300, China

**Keywords:** cider quality, *Wickerhamomyces anomalus*, *Saccharomyces cerevisiae*, co-inoculation

## Abstract

Co-inoculation with *Saccharomyces cerevisiae* and non-*Saccharomyces* yeasts is an effective method to improve the flavor of cider. *Wickerhamomyces anomalus*, known for its high ester production capacity, was evaluated in combination with *S. cerevisiae* to identify optimal mixed yeast inoculants for improved sensory characteristics. Three *W. anomalus* strains and three inoculation ratio attributes (1:5, 1:1, and 5:1) were tested to assess their impact on the physicochemical indices and sensory attributes of cider. All the strains used as starters developed fermentation-producing ciders with alcoholic degrees between 6.22 and 6.36 (% *v*/*v*). Co-inoculation with *W. anomalus* resulted in significantly higher ester, volatile acid, and higher alcohol levels compared to those of *S. cerevisiae* monocultures, increasing the complexity of fruity and floral aromas. Furthermore, the proportion of *W. anomalus* strains in the inoculations was positively correlated with increased aromatic esters and higher alcohols. The Sc–Wa (1:5) cider showed the highest contents of ethyl ethanoate and 3-methylbutan-1-ol, contributing to a nail polish-like aroma. Sc–Wa (1:1) yielded a higher aromatic diversity than did Sc–Wa (5:1), suggesting that co-inoculation with a ratio of 1:1 may provide an effective fermentation strategy for cider aroma enhancement. These findings offer valuable insights into how non-*Saccharomyces* yeasts can be effectively applied in cider co-fermentation, providing a foundation for their future use in industrial applications.

## 1. Introduction

Cider, a low-alcohol beverage typically containing approximately 1.2–8.5% alcohol by volume, is produced from the fermentation of apple juice [1]. The sensory complexity of ciders is influenced by multifaceted variables, including apple cultivars, ripeness, yeast strains, and fermentation techniques [2]. *Saccharomyces cerevisiae* has been predominantly used for cider production, primarily because of its remarkable metabolic efficiency in converting sugar substrates into alcohols [3]. To more effectively control the fermentation process and achieve more consistent results, the use of *S. cerevisiae* starter cultures during the alcoholic fermentation of apple juice has become a common approach in modern cider production [4]. However, ciders frequently exhibit poor aromas and sour flavors, a limitation that has substantially constrained the potential qualitative expansion and market diversification.

Non-*Saccharomyces* yeasts, with milder fermentation and richer aromas, offer potential solutions for these challenges [5]. Among them, *Wickerhamomyces anomalus* stands out for its exceptional physiological resilience and capacity to proliferate under challenging environmental conditions, including low temperatures, acidic pH gradients, and elevated alcohol concentrations [6]. This yeast has been reported to produce glycosidases, enzymes that release latent aromatic precursors from grape matrices, creating complex fruity and floral characteristics, along with volatile compounds that enhance the sensory complexity of fruit-based fermented beverages [7]. *W. anomalus* also generates acetate esters, such as ethyl ethanoate and 3-methylbutyl ethanoate, at higher levels than does *S. cerevisiae* [8]. Strategic co-inoculation of *S. cerevisiae* and *W. anomalus* has been empirically demonstrated to substantially improve the aromatic sophistication of diverse fruit wines, including kiwi [9], cider [10], and blueberry [11].

Over the past five years, researchers have increasingly experimented with non-*Saccharomyces* yeasts, such as *Pichia kudriavzevii* [12], *Torulaspora delbrueckii* [13], *Zygosaccharomyces bailii* [14], *Starmerella bacillaris* [14], *Rhodotorula mucilaginosa* [15], and *Debaryomyces hansenii* [15], either alone or in co-fermentation with *S. cerevisiae*, to enhance the sensory quality of cider. While these studies have partially addressed the issue of excessive acidity in cider, insufficient investigation into the optimal inoculation ratios for co-fermentation has led to delayed fermentation or undesirable flavors, posing challenges to industrial production. Additionally, certain fruit wines produced through the co-inoculation of *S. cerevisiae* and *W. anomalus* have shown elevated concentrations of acetate esters (e.g., ethyl ethanoate > 150 mg/L), resulting in undesirable sensory aberrations and compromised overall organoleptic quality [16]. Therefore, precise control of ratios of *W. anomalus* and *S. cerevisiae* during co-inoculation is critical to avoid fermentation delays and flavor deviations and ensure sensory integrity.

To address these challenges, we evaluated three *W. anomalus* strains and tested ten inoculation strategies using *S. cerevisiae* for cider fermentation. We analyzed the effects of varying inoculation ratios on the physicochemical characteristics, organic acid profiles, volatile compound compositions, and sensory attributes. This study contributes to the development of non-*Saccharomyces* yeast starters designed to enhance cider quality, focusing on the efficient management of fermentation using *W. anomalus* and optimized inoculation protocols.

## 2. Results and Discussion

### 2.1. Fermentation Kinetics

**Figure 1:**
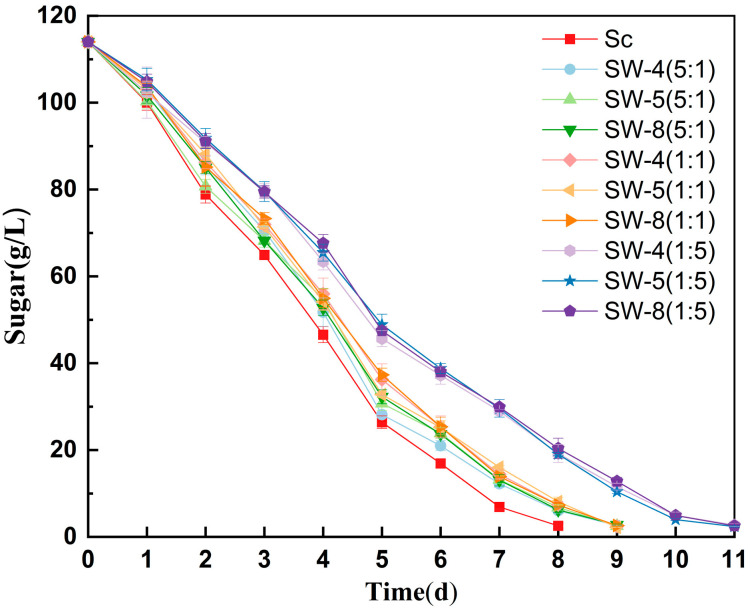
Fermentation kinetics (as sugar consumption) of 10 treatments.

**Figure 2:**
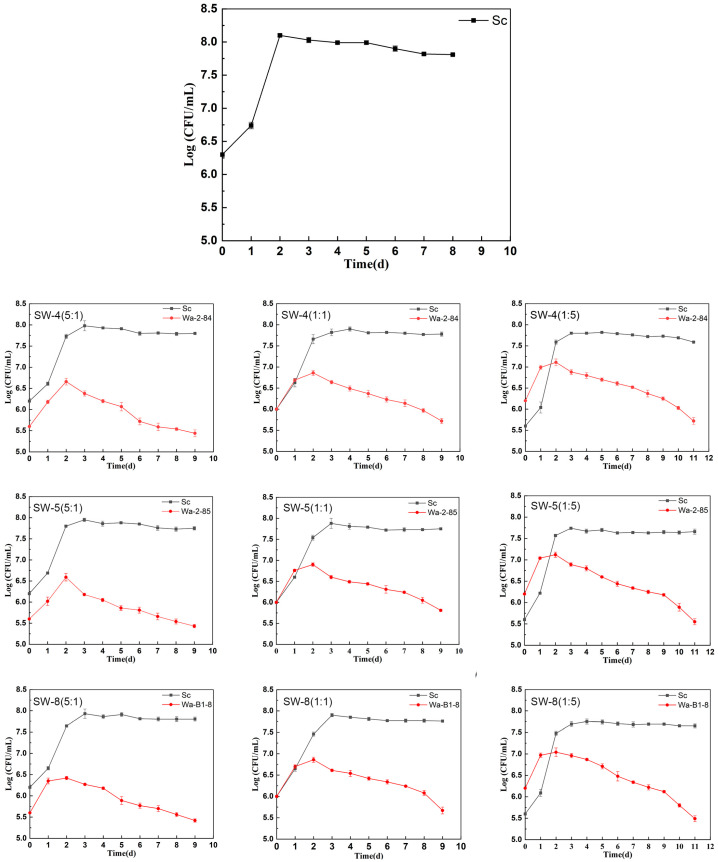
Biomass evolution (as log CFU ml^−1^) of yeast during fermentations with different inoculation treatments.

The effects of different inoculation ratios of *W. anomalus* and *S. cerevisiae* on cider fermentation were assessed, with all groups completing fermentation within 11 days (Figure 1). The duration of cider fermentation varied across *W. anomalus* strains, but statistical analysis revealed that these differences were not significant; however, the inoculation ratio had a more significant impact on the overall process (Figure 2). Higher proportions of *W. anomalus* extended the fermentation duration, with Sc–Wa ratios of 5:1 and 1:1 prolonging the process by 1 d, whereas a 1:5 ratio extended it by 3 d compared to that of the single culture fermentation of *S. cerevisiae* CECA. These findings suggest an antagonistic interaction, wherein *W. anomalus* populations decline earlier during co-inoculation, potentially delaying fermentation [17,18].

In all strategies, *W. anomalus* reached a peak concentration of 10^6^–10^7^ CFU/mL on day 2 before declining; however, it remained detectable throughout fermentation. The decline in *W. anomalus* was associated with ethanol production, competition for limited nutrients, and interactions with *S. cerevisiae* [19]. In single-culture fermentation, *S. cerevisiae* CECA rapidly peaked at 10^8^ CFU/mL on day 2, whereas in co-fermentation, the concentration peak was delayed until day 3, consistently remaining higher than that of *W. anomalus* during the middle and later stages of fermentation. This phenomenon may be attributed to the high resistance of *S. cerevisiae* to alcohol. Despite *S. cerevisiae* consistently outcompeting *W. anomalus*, its peak populations were lower in co-cultures than in single cultures, suggesting that *W. anomalus* inhibits the growth of *S. cerevisiae* [18].

### 2.2. Physicochemical Parameters

All strains completed fermentation and maintained residual sugar concentrations below 3 g/L (Figure 1). The physicochemical characteristics of the samples are presented in Table 1. The alcohol, malic, lactic, and citric acid contents remained relatively stable across treatments, unaffected by the inoculation ratio or strain variations. Ethanol production during co-fermentation remained consistent between cultures. All strains used as starters successfully fermented, resulting in ciders with alcohol contents ranging from 6.22% to 6.36% (*v*/*v*). On average, the alcohol content was 6.31% (*v*/*v*) for SW-4, 6.24% (*v*/*v)* for SW-5, and 6.35% (*v*/*v*) for SW-8. There are no statistically significant differences in ethanol production between the yeast inoculation modalities.

The pH increased with higher *S. cerevisiae* inoculation ratios, whereas no significant differences were observed among the various strains at the same inoculation ratio. This finding aligns with those of previous studies, indicating that an increase in pH correlates with a significant decrease in titratable acidity [20]. Furthermore, in wine fermentation, the co-fermentation of *W. anomalus* and *S. cerevisiae* results in lower acidity than that of monocultures of *S. cerevisiae* [21].

Acidity is a crucial factor determining the sensory characteristics of cider [22]. The evaluation of organic acid levels revealed the presence of seven organic acids in the cider. Among these, malic acid was identified as the dominant, accounting for 46.3% to 49.4% of the total organic acids, followed by citric acid, which constituted 17.6% to 19.4% of the total organic acids. The volatile acid content of *S. cerevisiae* (0.27 g/L) was lower than that from co-inoculation, with the Sc–Wa (1:5) exhibiting the highest levels of volatile acids.

All Sc–Wa treatments attenuated cider acidification, resulting in a higher pH and lower titratable acidity (TA) compared to that of *S. cerevisiae*. These changes in organic acid profiles highlight the influence of *W. anomalus* on cider acidity.

### 2.3. Volatile Profiles

A total of 22 volatile compounds were identified in the fermented apple juice samples, comprising yeast-derived metabolites, including nine esters, one aldehyde, nine higher alcohols, and three acids (Table 2). These compounds have been quantified and recognized as the primary contributors to cider aroma [15].

**Figure 3:**
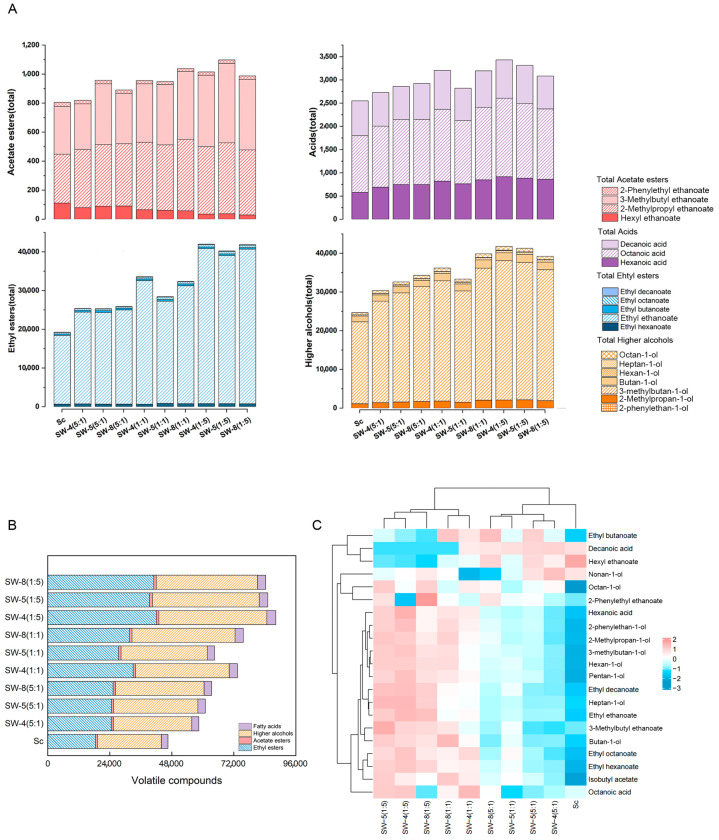
Volatile compounds of ciders: (**A**) total concentration (µg/L); (**B**) classification (µg/L); and (**C**) heatmap.

Esters (19,269–43,005 μg/L) were the most abundant volatile compound, comprising five ethyl esters (ethyl ethanoate, ethyl butanoate, ethyl hexanoate, ethyl octanoate, and ethyl decanoate) and four acetates (3-methylbutyl ethanoate, hexyl ethanoate, and 2-phenylethyl ethanoate). Generally, esters positively contribute to wine aroma and are considered major contributors to sweet and fruity odors [20]. Although some esters might be present in apple juice prior to fermentation, the majority are formed through the enzymatic activity of yeast and the synthesis or degradation of fatty acids during fermentation [23,24]. Compared with CECA alone, co-inoculation with *W. anomalus* and *S. cerevisiae* significantly increased the ester content, enhancing the aroma of the cider, which is consistent with the results of previous reports [25]. Ethyl ethanoate, the most abundant ester (Figure 3), is critical for cider flavor, where low concentrations (<150 mg/L) impart fruity and pleasant aromas reminiscent of pineapple, pear, and banana, and elevated concentrations (>200 mg/L) can lead to undesirable flavors in cider [20,24,26,27]. We observed a correlation between increased proportions of *W. anomalus* and higher ethyl ester content. Specifically, SW-4 (1:5), SW-5 (1:5), and SW-8 (1:5) produced ethyl ethanoate levels over two-fold higher than those of CECA alone, with SW-4 producing the highest concentration among all tested strains. Despite this increase, the ethyl ethanoate content in all the co-inoculated ciders remained below the deemed faulty levels. Additionally, among other minor esters, ethyl butyrate, ethyl decanoate, 2-phenylethyl ethanoate, and hexyl ethanoate constituted the quantitatively dominant fractions of volatile compounds in cider, which is consistent with the results of previous studies [28]. The changes exhibited by ethyl hexanoate, ethyl octanoate, and ethyl decanoate were similar to those of ethyl ethanoate across the various treatments, with the highest content observed at the Sc–Wa (1:5) inoculation ratio. In contrast, ethyl butanoate displayed the highest content at the Sc–Wa (5:1) ratio. Non-*Saccharomyces* yeasts influence the production of ethyl esters in fruit wine, potentially due to the presence of secretases typically absent in *S. cerevisiae*, such as α-L-arabinofuranosidase, β-glucosidase, polygalacturonase, cellulase, and protease [25,29].

Acetate esters are generated by the reaction of acetyl-CoA with higher alcohols produced by the degradation of amino acids or carbohydrates [30]. In co-fermented apple juice, the 2-methylpropyl ethanoate and 3-methylbutyl ethanoate content increased, whereas the hexyl ethanoate concentration decreased compared to that of CECA. Additionally, there was no significant correlation between a higher percentage of *W. anomalus* inoculation and the product content. The concentration of acetate was found to be more affected by enzyme activity than by substrate utilization when compared to the results for the ethyl esters, suggesting differences in acetyl transferases between yeast stains [31].

Alcohols constituted the second-largest group of volatile compounds (by content) in the apple cider samples. Most higher alcohols are produced through yeast fermentation and serve as important precursors for esters and key contributors to cider aroma [32]. When the concentration of higher alcohols is below 300 mg/L, it imparts a pleasant aroma to wine [33]; however, an excessive content of higher alcohols can negatively affect wine flavor [34]. Co-fermentation produced higher alcohol levels than did single-culture CECA; however, all treatments remained within acceptable ranges (<8.5%). This phenomenon has also been reported in previous studies of wines fermented with a mixture of *L. thermotolerans* and *S. cerevisiae* [31]. The most abundant higher alcohol (comprising over 80% of total higher alcohols) was 3-methylbutan-1-ol in SW-4 (1:5) (36,013 μg/L), followed by butan-1-ol. This compound has been reported as the most abundant alcohol in ciders [23,35]. SW (1:5) contained more than double the amount of hexan-1-ol compared to that in CECA, which has been shown to enhance the aroma with notes of green grass and alcohol in fruit wine [33]. The levels of 1-decanal showed little variation among the ten cider groups. The production of 2-Methylpropan-1-ol, 3-methylbutan-1-ol, and 2-phenylethan-1-ol during fermentation occurs through the conversion of valine, leucine, and phenylalanine via the Ehrlich pathway during fermentation [36]. Sc–Wa (5:1), Sc–Wa (1:1), and SW-8 showed higher levels of total higher alcohols. The content was the lowest in Sc–Wa (1:5).

Fatty acids are significant aromatic components produced directly from apples. Their concentrations depend on various factors, including the apple cultivar, harvest time, and alcoholic fermentation process [37]. Three fatty acids, hexanoic, octanoic, and decanoic acids, were identified in the apple cider, with octanoic acid exhibiting the highest concentration. The highest fatty acid levels were observed in the Sc–Wa (1:5) treatments. These acids likely have a minimal impact on the overall flavor profile due to their high odor-detection thresholds [33].

Figure 3B compares the total concentration of volatile flavor compounds in the cider samples, with co-inoculation significantly enhancing the volatile content compared to single-culture CECA. Volatile flavor components across the cider samples exhibited significant variations in both species and content, which is likely attributable to the different species of non-*Saccharomyces* yeast [14]. Notably, the *W. anomalus* inoculated samples contained a greater number of volatile compounds than did CECA, suggesting that microorganisms generated a substantial number of metabolites during fermentation, which is consistent with the results of previous research [8]. As shown in Figure 3A, the total concentrations of esters, alcohols, and acids varied among cider samples. Specifically, the total concentration of esters in CECA was markedly lower than that in other cider samples. A heatmap analysis (Figure 3C) further illustrates differences in volatile profiles, highlighting the strong contribution of *W. anomalus* to ester and higher alcohol production.

The concentration of volatile substances generated during fermentation, influenced by the inoculation ratio of Sc–Wa, exhibited a relatively minor correlation with the different *W. anomalus* strains.

### 2.4. Multivariate Analysis of the Chemical Parameters

**Figure 4:**
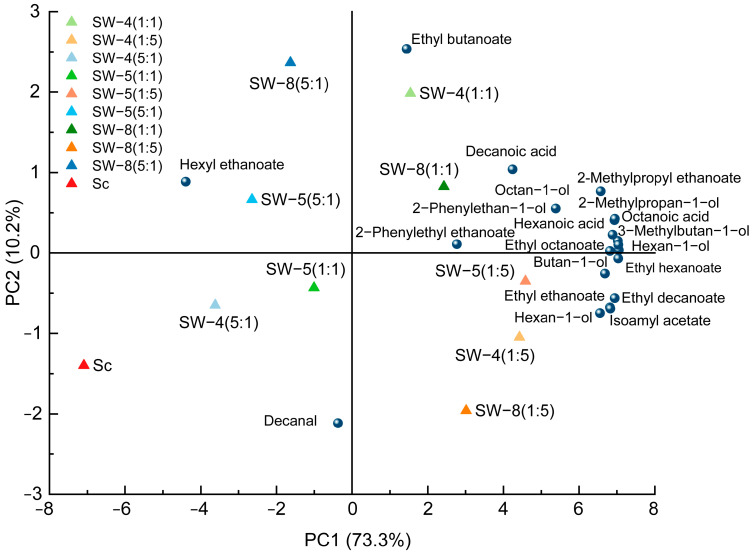
PCA of the oenological properties of ciders.

The variability in the aromatic compounds of cider was visualized through principal component analysis (PCA) (Figure 4). The first two principal components collectively accounted for 83.5% of the total variance. PCA revealed a clear separation between the CECA monoculture and co-inoculation treatments along PC1, which accounted for 73.3% of the total explained variance. The majority of volatile compounds clustered in the first and fourth quadrants of the negative area of PC1, with strong associations to the SW-4 (1:5), SW-5 (1:5), and SW-8 (1:5) treatments. This suggests that co-inoculation enhanced the intensity of the apple cider aroma, with the effect becoming more pronounced as the proportion of *W. anomalus* inoculation increased. Ciders fermented with CECA exhibited higher concentrations of hexyl ethanoate, titratable acidity, and pyruvic acid. Differentiation among the various inoculation ratios in the mixed fermentations was driven by increases in ethyl esters, higher alcohols (excluding nonan-1-ol), and fatty acids. Additionally, co-fermented apple ciders with different *W. anomalus* strains showed distinct separation along PC2. Overall, co-inoculation with *W. anomalus* and *S. cerevisiae* resulted in increased pH, acetic acid, and volatile compound contents in the cider.

### 2.5. Sensory Profiles of Ciders

**Figure 5:**
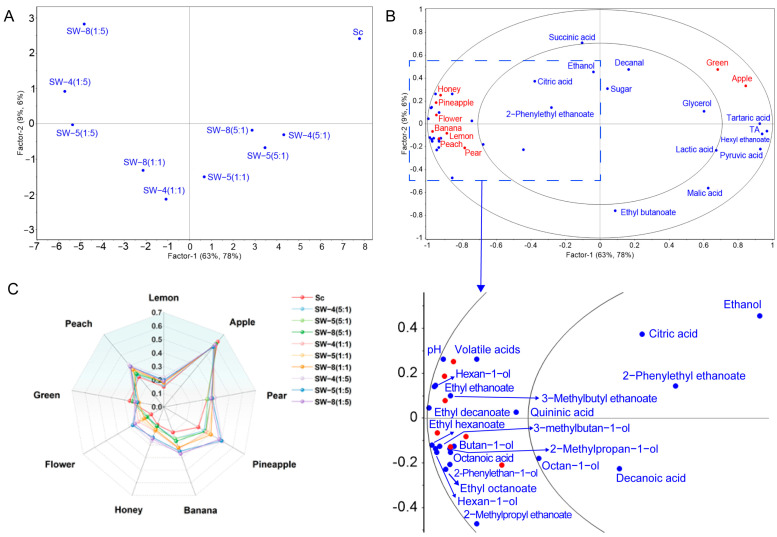
PLS regression analysis of sensory profiles of ciders: (**A**) yeast treatments; (**B**) configuration of sensory profiles; (**C**) modified frequency (*MF*) values of aroma characteristics in different cider fermentations.

The PLSR multivariate statistical analysis method was applied to establish a regression model linking sensory characteristics and chemical components [5]. The first two components effectively distinguished the yeast treatments, accounting for 72% and 84% of the variation in the chemical and sensory profiles, respectively (Figure 5A,B). The first component separated the *S. cerevisiae* control from the co-inoculation treatments, with further divergence observed based on different inoculation ratios. This separation along the first component was influenced by increases in ethyl esters and higher alcohols, in contrast to higher hexyl ethanoate and acid contents. The SW-4 (1:1), SW-5 (1:1), and SW-8 (1:5) treatments were distinctly separated from the remaining co-inoculation treatments in the second component.

Figure 5C illustrates the *MF* values of the nine aroma descriptors: citrus (lemon), kernel fruit (apple and pear), drupe fruit (peach), tropical fruit (pineapple and banana), sweet (honey), floral, and green. All descriptors, except for kernel fruit and green, were significantly enhanced with higher proportions of *W. anomalus* inoculation (*p* < 0.05). The highest concentrations of ethyl ethanoate and 3-methylbutan-1-ol were found in ciders with Sc–Wa (1:5), and these ciders exhibit a chemical odor similar to nail polish, which may be related to the content of ethyl ethanoate. Excessively high levels of ethyl ethanoate can impart a chemical or nail polish-like smell to the fruit wine [27]. However, in our analysis, the detected level of ethyl ethanoate did not exceed the threshold for adverse effects (>200 mg/L) (Table 2); this suggests that factors other than the concentration may be influencing the sensory perception of ethyl ethanoate; thus, the specific reasons for the adverse sensory perception still need to be further explored. Overall, the tropical fruit and floral traits were notably enhanced by the *W. anomalus* strain. The co-inoculation process enhanced the tropical fruit and floral aromas, which is consistent with results from previous studies [9].

## 3. Materials and Methods

### 3.1. Apple and Yeast Strains

Red Fuji apples (*Malus domestica*) were procured from an orchard in Qin’an County, Shanxi Province, China. Initial apple juice produced from these apples yielded 114 g/L glucose (approximately 11° Brix), 3.2 g/L total acids, and a pH of 3.81.

Four yeast strains were used: *S. cerevisiae* CECA, supplied by Angel Yeast Company (Angel, Yichang, China), and three *W. anomalus* strains (Wa-2-84, Wa-2-85, and Wa-B18) isolated from mutant strains generated via ARTP (atmospheric room temperature plasma) mutagenesis. All the strains were preserved at the College of Enology, Northwest A&F University (Xianyang, China).

### 3.2. Fermentation Setup

Following peeling, pitting, and juicing, 120 mg/L of potassium metabisulphite was immediately added to the apple juice to prevent oxidative reactions and inhibit microbial activity. After 30 min, 0.1 g/kg of pectinase (RF, AB Enzymes GmbH, Darmstadt, Germany) was added to the juice, which was then cold-stabilized and stored at 4 °C for 48 h to clarify it for fermentation.

Fermentation was conducted in triplicate using sterile glass bottles containing 500 mL of apple juice at 18 °C under static conditions. The clarified juice was inoculated using the strategies described in Table 3. *W. anomalus* strains (Wa-2-84, Wa-2-85, and Wa-B18) and *S. cerevisiae* CECA were simultaneously inoculated at 2 × 10^6^ CFU/mL, respectively. Prior to inoculation, the yeast strains underwent a precultivation process in a liquid YPD medium, which comprised yeast extract (10 g/L), peptone (20 g/L), and glucose (20 g/L)(AOBOX, Beijing, China). This precultivation step took place at a controlled temperature of 28 °C and lasted for a period of 48 h. Afterwards, the culture broth was centrifuged at 4500× *g* for 10 min to collect the yeast pellets. Subsequently, the yeast pellets were washed twice with sterile normal saline (0.9%). After that, the yeast was resuspended in sterile 0.9% sodium chloride solution and prepared for use in the subsequent fermentation. The reducing sugar concentrations were monitored daily (Section 3.3). Fermentation was considered complete when reducing sugar levels stabilized over two consecutive days, at which point the yeast was separated from the medium via centrifugation at 10,000 rpm for 10 min. The resulting supernatant was stored under quiescent conditions at 4 °C in a light-protected, well-ventilated environment for subsequent analysis.

### 3.3. Determination of Physicochemical Parameters and Volatile Compounds

The total sugar was titrated using Fehling reagent [38]. Yeast growth was monitored through plate counting on Wallerstein nutrient agar (WL) medium (Haibo Bio Ltd., Qingdao, China) every 24 h. Prior to counting, the WL plates were incubated at 28 °C for 72 h. Titratable acidity (TA), pH, and ethanol concentrations were evaluated following the established international protocols outlined in the OIV (International Organization of Vine and Wine) Compendium (2024 edition) for wine and must analysis [39]. The glycerol content was quantified using a Y15 Automatic Wine Analyzer (Biosystems, Barcelona, Spain). Organic acids were analyzed using a high-performance liquid chromatograph (HPLC), 260 Infinity II instrument (Agilent Technologies, Santa Clara, CA, USA) equipped with an Aminex HOX-87H ion-exchange column (300 mm × 7.8 mm) and a photo-diode array detector (PDA, Shimadzu, Japan) under the following conditions: detection at 214 nm, column temperature of 60 °C, and a mobile phase of 5 mmol/L H₂SO₄, with a flow rate of 0.6 mL/min.

### 3.4. Determination of Volatile Compounds

The volatile compounds were extracted using headspace solid-phase microextraction (HS-SPME) [40]. For each extraction, 5 mL of cider sample was pipetted into a vial and 1 g of sodium chloride and 10 μL 4-methyl-2-pentanol (1.0 g/L, added as an internal standard) were added. For extraction, an activated SPME fiber (DVB/C-WR/PDMS) (Agilent Technologies, Santa Clara, CA, USA) was employed at 250 rpm, 40 °C, for 30 min. The volatiles were analyzed by gas chromatography–mass spectrometry (GC–MS) using a Trace 1310 GC coupled to an ISQ-LT MS (Thermo Scientific, Waltham, MA, USA) and an HP-INNOWAX column (60 m × 0.25 mm × 0.25 μm; Agilent J & W, Santa Clara, CA, USA). The volatiles were carried by helium at 1 mL/min. The temperature of the injector and transfer line was 250 °C. The program is as follows: initial temperature 50 °C hold for 1 min, raised to 220 °C at 3 °C/min, and hold for 5 min. MS program: electron impact ionization mode, ion source temperature 250 °C, mass range of *m*/*z* 250–350, scanned at 0.2 s intervals. The volatiles were identified by comparing their retention times and mass spectra with those of pure standards using the NIST20 library. Standard calibration curves were constructed using volatile compound standards in a synthetic wine medium (14% *v*/*v* ethanol, 5 g/L tartaric acid, pH 3.8) [41]. Agilent ChemStation (Agilent Technologies, Santa Clara, CA, USA) was used to quantify the volatile compounds. The concentrations of the compounds were calculated using calibration curves, following a method previously described in Ref. [42].

### 3.5. Sensory Analysis

Sensory evaluation was performed in duplicate by a trained tasting panel of seven females and seven males, who underwent a one-month training program with a 54-aroma kit (Le Nez du Vin^®^, Rennes, France). The panelists were tested every six days during training, ensuring an accuracy exceeding 95% in regards to aroma identification. Evaluations were conducted in a tasting room maintained at 23 °C, using approximately 30 mL of cider served at 15 °C in black wine glasses, presented in a randomized order.

The panelists followed a structured procedure for sensory analysis, assessing the static aroma for 5–8 s, followed by agitation and reassessment for an additional 5–10 s. They described the aroma using 4-6 terms from the aroma kit and rated its intensity on a five-point scale, where 1 = weak, 2 = slightly weak, 3 = medium, 4 = slightly intense, and 5 = intense. Final aroma characteristics were expressed as the modified frequency (*MF*, %) [43], as follows:

$$MF\%=\sqrt{F\%I\%}$$

where *F%* is the detection frequency for each aromatic attribute, and *I%* is average intensity.

### 3.6. Statistical Analysis

Data were computed in Excel 2024 and are expressed as mean ± standard deviation. Significant differences were identified using one-way ANOVA and Duncan’s multi-range test, with a significance level set at *p* < 0.05 (SPSS 26.0, SPSS Inc., Chicago, IL, USA). PCA was performed using XLSTAT 2025 (Addinsoft SARL), with data normalized using the z-score method, to compare the volatile characteristics of wines fermented using the different treatments. Partial least squares regression (PLS-R) was conducted using Unscrambler X software (Version 10.3; CAMO Software, Oslo, Norway). Graphs were generated using Origin 2024 software (OriginLab Corporation, Northampton, MA, USA).

## 4. Conclusions

This paper corroborates the work obtained by other authors and complements the significant differences among different inoculation ratios in mixed-fermentation. Three *W. anomalus* strains, Wa-2-84, Wa-2-85, Wa-B1-8, along with *S. cerevisiae* CECA, were used to ferment Fuji apple juice under static conditions, producing the following findings. Co-inoculation of *W. anomalus* and *S. cerevisiae* resulted in extended fermentation times. The alcohol and malic, lactic, and citric acid contents remained relatively stable across treatments, unaffected by the inoculation ratio or strain variations. All Sc–Wa treatments attenuated cider acidification, resulting in a higher pH and lower titratable acidity compared to those from *S. cerevisiae*.

Among all inoculation strategies, co-inoculation with *W. anomalus* prolonged the fermentation process compared to that of CECA. Specifically, the SW (1:5) ratio exhibited the most significant delay (2 days), whereas both SW (1:1) and SW (5:1) ratios resulted in a 1-day extension. Furthermore, the production of aroma compounds during fermentation was influenced by the inoculation ratio of Sc–Wa, with a minor impact from different *W. anomalus* strains. Co-inoculation increased the production of esters and higher alcohols, enhancing the “fruity” and “floral” aroma profiles of the cider. Notably, the SW (1:5) group developed undesirable off-odors during sensory evaluation, and the causes of this phenomenon require further investigation. Considering that the SW (5:1) group showed inferior volatile compound production relative to that of SW (1:1), the 1:1 inoculation ratio was identified as the optimal protocol, balancing fermentation efficiency and aroma profile enrichment. The performance of different treatments demonstrates that the inoculation ratio of non-*Saccharomyces* yeasts during co-inoculation is a crucial factor that should not be overlooked. These findings highlight the potential of *W. anomalus* in enhancing the quality of cider when co-inoculated with *S. cerevisiae*. For industrial applications, we suggest a preliminary screening of inoculation ratios to target specific aroma profiles, as this could optimize the fermentation process and enhance the overall quality of the final product.

This study provides valuable insights into the conditions under which non-*Saccharomyces* yeasts can effectively be used in the co-inoculation of ciders, laying the groundwork for their broader application. Future studies on industrial-scale fermentation and sensory tasting should be undertaken to assess the effects of *W. anomalus* strains on cider mouthfeel. Furthermore, investigating the metabolic pathways and regulatory mechanisms of different *W. anomalus* strains during co-inoculation will be essential for optimizing their role in cider fermentation.

## Figures and Tables

**Table 1:** Physicochemical parameters of ciders fermented with different yeast inoculation modalities.

	Yeast Treatment									
	SC	SW-4 (5:1)	SW-5 (5:1)	SW-8 (5:1)	SW-4 (1:1)	SW-5 (1:1)	SW-8 (1:1)	SW-4 (1:5)	SW-5 (1:5)	SW-8 (1:5)
Residual sugar (g/L)	2.53 ± 0.09 ^ab^	2.60 ± 0.21 ^a^	2.52 ± 0.24 ^ab^	2.71 ± 0.23 ^a^	2.45 ± 0.16 ^ab^	2.19 ± 0.11^b^	2.61 ± 0.16 ^a^	2.70 ± 0.20 ^a^	2.38 ± 0.16 ^ab^	2.53 ± 0.09 ^ab^
Glycerol (g/L)	3.20 ± 0.12 ^ab^	3.10 ± 0.09 ^bc^	3.16 ± 0.10 ^ab^	3.33 ± 0.14 ^a^	2.78 ± 0.05 ^d^	2.71 ± 0.17 ^d^	3.24 ± 0.03 ^ab^	2.71 ± 0.09 ^d^	2.88 ± 0.08 ^d^	2.92 ± 0.10 ^cd^
Ethanol (% *v*/*v*)	6.35 ± 0.07 ^a^	6.30 ± 0.07 ^a^	6.22 ± 0.11 ^a^	6.35 ± 0.13 ^a^	6.29 ± 0.08 ^a^	6.24 ± 0.13 ^a^	6.36 ± 0.20 ^a^	6.33 ± 0.16 ^a^	6.27 ± 0.18 ^a^	6.34 ± 0.07 ^a^
pH	3.69 ± 0.01 ^e^	3.71 ± 0.01 ^de^	3.69 ± 0.02 ^e^	3.71 ± 0.02^cd^	3.73 ± 0 ^bc^	3.72 ± 0.02 ^bcd^	3.74 ± 0.01^b^	3.79 ± 0.01 ^a^	3.79 ± 0.01 ^a^	3.79 ± 0.01 ^a^
TA(g/L)	4.53 ± 0.03 ^a^	4.40 ± 0.15 ^abc^	4.46 ± 0.07 ^ab^	4.45 ± 0.19 ^ab^	4.31 ± 0.03 ^bcd^	4.39 ± 0.10 ^abc^	4.30 ± 0.03 ^bcd^	4.17 ± 0.05 ^d^	4.30 ± 0.14 ^bcd^	4.21 ± 0.03 ^cd^
Volatile acids (g/L)	0.27 ± 0.01 ^d^	0.27 ± 0.02 ^cd^	0.29 ± 0.01 ^bcd^	0.28 ± 0.01 ^bcd^	0.30 ± 0 ^bc^	0.28 ± 0.01 ^bcd^	0.31 ± 0.02^b^	0.30 ± 0.01 ^b^	0.33 ± 0 ^a^	0.34 ± 0.02 ^a^
Malic acid (g/L)	2.34 ± 0.06 ^a^	2.38 ± 0.18 ^a^	2.32 ± 0.12 ^a^	2.38 ± 0.09 ^a^	2.39 ± 0.14 ^a^	2.32 ± 0.07 ^a^	2.28 ± 0.10 ^a^	2.20 ± 0.08 ^a^	2.33 ± 0.17 ^a^	2.17 ± 0.15 ^a^
Pyruvic acid (g/L)	0.34 ± 0.04 ^a^	0.34 ± 0.01 ^ab^	0.34 ± 0.02 ^a^	0.34 ± 0.02 ^a^	0.28 ± 0 ^c^	0.27 ± 0.02 ^c^	0.29 ± 0.03 ^bc^	0.21 ± 0.01^d^	0.22 ± 0.01^d^	0.22 ± 0^d^
Acetic acid (g/L)	0.35 ± 0.04 ^b^	0.36 ± 0.02 ^b^	0.37 ± 0.03 ^b^	0.37 ± 0.04 ^b^	0.36 ± 0.03 ^b^	0.35 ± 0.02 ^b^	0.37 ± 0.04 ^b^	0.46 ± 0.02 ^a^	0.43 ± 0.02 ^a^	0.46 ± 0.01 ^a^
Lactic acid (g/L)	0.14 ± 0.01 ^a^	0.15 ± 0.02 ^a^	0.14 ± 0.01 ^a^	0.15 ± 0.03 ^a^	0.14 ± 0.01 ^a^	0.13 ± 0.01 ^a^	0.15 ± 0.02 ^a^	0.13 ± 0.02 ^a^	0.14 ± 0.02 ^a^	0.13 ± 0.01 ^a^
Citric acid (g/L)	0.90 ± 0.07 ^a^	0.87 ± 0.09 ^a^	0.93 ± 0.05 ^a^	0.95 ± 0.01 ^a^	0.89 ± 0.02 ^a^	0.87 ± 0.05 ^a^	0.93 ± 0.09 ^a^	0.92 ± 0.04 ^a^	0.94 ± 0.02 ^a^	0.95 ± 0.03 ^a^
Succinic acid (g/L)	0.55 ± 0.05 ^ab^	0.53 ± 0.02 ^ab^	0.55 ± 0.06 ^ab^	0.58 ± 0.03 ^a^	0.48 ± 0.01 ^b^	0.48 ± 0.03^b^	0.53 ± 0.07 ^ab^	0.54 ± 0.06 ^ab^	0.56± 0.02 ^ab^	0.59 ± 0.03 ^a^
Quininic acid (g/L)	0.15 ± 0.01 ^d^	0.17 ± 0 ^bc^	0.18 ± 0.01 ^abc^	0.18 ± 0 ^ab^	0.16 ± 0 ^cd^	0.17 ± 0.01 ^bc^	0.19 ± 0.01 ^a^	0.18 ± 0 ^ab^	0.19 ± 0 ^ab^	0.19 ± 0.02 ^a^

Note: The values are expressed as the mean ± standard deviation, based on three parallel experiments; titratable acidity (TA) expressed as malic acid. In the same row, lowercase letters (a–e) are used to indicate results derived from the least significant difference test (*p* < 0.05, Duncan).

**Table 2:** The volatile compound of ciders fermented with different yeast inoculation modalities.

Compounds (µg/L)	Yeast Treatment									
	SC	SW-4 (5:1)	SW-5 (5:1)	SW-8 (5:1)	SW-4 (1:1)	SW-5 (1:1)	SW-8 (1:1)	SW-4 (1:5)	SW-5 (1:5)	SW-8 (1:5)
Ethyl ethanoate	17,756 ± 441 ^f^	23,686 ± 732 ^e^	23,663 ± 697 ^e^	24,292 ± 1551 ^de^	31,928 ± 1704 ^c^	26,388 ± 669 ^d^	30,456 ± 939 ^c^	40,860 ± 1176 ^a^	38,272 ± 1305 ^b^	39,940 ± 969 ^ab^
Ethyl butanoate	381 ± 21 ^e^	433 ± 24 ^bcd^	481 ± 8 ^ab^	493 ± 17 ^a^	467 ± 17 ^abc^	434 ± 7 ^bcd^	488 ± 18 ^a^	406 ± 20 ^de^	428 ± 5 ^cde^	396 ± 5 ^de^
Ethyl hexanoate	132 ± 3 ^f^	173 ± 8 ^e^	178 ± 7 ^de^	192 ± 8 ^d^	228 ± 10 ^b^	210 ± 6 ^c^	224 ± 12 ^bc^	255 ± 5 ^a^	249 ± 4 ^a^	245 ± 3 ^a^
Ethyl octanoate	170 ± 14 ^c^	207 ± 21 ^bc^	227 ± 21 ^b^	227 ± 7 ^b^	321 ± 20 ^a^	288 ± 8 ^a^	297 ± 8 ^a^	341 ± 17 ^a^	320 ± 20 ^a^	305 ± 25 ^a^
Ethyl decanoate	47 ± 4 ^d^	69 ± 3 ^c^	71 ± 5 ^c^	80 ± 4 ^c^	108 ± 12 ^b^	102 ± 6 ^b^	111 ± 9 ^b^	147 ± 8 ^a^	146 ± 8 ^a^	138 ± 9 ^a^
Ʃ Ethyl esters	18,487 ± 445 ^f^	24,567 ± 681 ^e^	24,620 ± 670 ^e^	25,285 ± 1516 ^de^	33,052 ± 1762 ^c^	27,423 ± 670 ^d^	31,576 ± 910 ^c^	42,009 ± 1140 ^a^	39,415 ± 1272 ^b^	41,025 ± 938 ^ab^
2-Methylpropyl ethanoate	337 ± 24^c^	402 ± 12^b^	425 ± 6 ^ab^	429 ± 20 ^ab^	465 ± 8 ^ab^	451 ± 4 ^ab^	492 ± 15 ^a^	465 ± 23 ^ab^	489 ± 22 ^a^	448 ± 41 ^ab^
3-Methylbutyl ethanoate	329 ± 11 ^e^	314 ± 23 ^e^	319 ± 7 ^e^	347 ± 13 ^e^	403 ± 17 ^d^	417 ± 8 ^cd^	469 ± 30 ^bc^	492 ± 21 ^b^	548 ± 25 ^a^	486 ± 14 ^b^
Hexyl ethanoate	111 ± 3 ^a^	79 ± 0 ^c^	89 ± 1 ^b^	91 ± 1 ^b^	65 ± 4 ^d^	61 ± 1 ^de^	58 ± 2 ^e^	35 ± 1 ^f^	37 ± 2 ^f^	29 ± 0 ^g^
2-Phenylethyl ethanoate	4 ± 0 ^a^	5 ± 0 ^a^	5 ± 1 ^a^	5 ± 1 ^a^	5 ± 0 ^a^	5 ± 0 ^a^	5 ± 0 ^a^	4 ± 0 ^a^	5 ± 0 ^a^	6 ± 0 ^a^
Ʃ Acetate esters	782 ± 37 ^d^	800 ± 35 ^d^	839 ± 13 ^d^	872 ± 33 ^cd^	938 ± 14 ^bc^	934 ± 5 ^bc^	1024 ± 46 ^ab^	996 ± 5 ^ab^	1079 ± 2 ^a^	969 ± 54 ^b^
2-Methylpropan-1-ol	1126 ± 51 ^f^	1394 ± 44 ^e^	1583 ± 112 ^de^	1720 ± 30 ^cd^	1835 ± 46 ^bc^	1528 ± 87 ^de^	2063 ± 115 ^ab^	2092 ± 44 ^a^	2122 ± 45 ^a^	1922 ± 119 ^abc^
Butan-1-ol	1497 ± 117 ^d^	1609 ± 56 ^cd^	1604 ± 148 ^cd^	1602 ± 78 ^cd^	1872 ± 36 ^abc^	1787 ± 28 ^bcd^	2126 ± 151 ^a^	2002 ± 46 ^ab^	2049 ± 57 ^ab^	1967 ± 77 ^ab^
3-methylbutan-1-ol	21,163 ± 1143 ^f^	26,231 ± 138 ^e^	28,199 ±771 ^d^	29,702 ± 150 ^cd^	31,044 ± 403 ^c^	28,750 ± 358 ^d^	34,097 ± 390 ^ab^	36,013 ± 681 ^a^	35,507 ± 233 ^ab^	33,821 ± 1094 ^b^
Hexan-1-ol	260 ± 19 ^f^	376 ± 4 ^e^	412 ± 13 ^d^	441 ± 2 ^cd^	493 ± 7 ^b^	460 ± 6 ^c^	567 ± 9 ^a^	595 ± 11 ^a^	593 ± 3 ^a^	565 ± 17 ^a^
Heptan-1-ol	44 ± 2 ^d^	53 ± 3 ^cd^	52 ± 2 ^cd^	55 ± 1 ^c^	69 ± 6 ^b^	58 ± 2 ^c^	72 ± 2 ^b^	93 ± 1 ^a^	92 ± 4 ^a^	86 ± 3 ^a^
Octan-1-ol	5 ± 0 ^c^	5 ± 0 ^ab^	5 ± 0 ^b^	5 ± 0 ^b^	6 ± 0 ^ab^	6 ± 0 ^ab^	6 ± 0 ^ab^	6 ± 0 ^ab^	6 ± 0 ^a^	6 ± 0 ^ab^
Nonan-1-ol	2 ± 0 ^d^	2 ± 0 ^d^	2 ± 0 ^d^	2 ± 0 ^d^	2 ± 0 ^b^	2 ±0 ^c^	2 ± 0 ^b^	2 ± 0 ^a^	2 ± 0 ^a^	2 ± 0 ^a^
2-phenylethan-1-ol	558 ± 38 ^e^	676 ± 16 ^b^	753 ± 21 ^c^	782 ± 17 ^c^	862 ± 19 ^b^	737 ± 13 ^c^	918 ± 21 ^ab^	976 ± 14 ^a^	940 ± 3 ^a^	854 ± 32 ^b^
Pentan-1-ol	ND	13 ± 1^c^	12 ± 0^c^	22 ± 2 ^a^	15 ± 1 ^bc^	16 ± 0 ^b^	16 ± 0 ^b^	14 ± 0 ^bc^	15 ± 0 ^bc^	16 ± 0 ^b^
Ʃ Higher alcohols	24,655 ± 1348 ^f^	30,358 ± 207 ^e^	32,622 ± 1064 ^d^	34,330 ± 272 ^cd^	36,198 ± 516 ^c^	33,344 ± 490 ^d^	39,866 ± 601 ^ab^	41,791 ± 786 ^a^	41,325 ± 281 ^ab^	39,237 ± 1306 ^b^
Hexanoic acid	579 ± 40 ^f^	691 ± 7 ^e^	748 ± 25 ^d^	747 ± 12 ^d^	821 ± 20 ^c^	763 ± 8 ^d^	849 ± 10 ^bc^	917 ± 9 ^a^	883 ± 8 ^ab^	861 ± 22 ^abc^
Octanoic acid	1220 ± 41 ^d^	1313 ± 16 ^c^	1398 ± 39 ^c^	1404 ± 29 ^c^	1547 ± 42 ^b^	1365 ± 22^c^	1561 ± 11 ^b^	1690 ± 24 ^a^	1610 ± 16 ^ab^	1516 ± 44 ^b^
Decanoic acid	750 ± 8 ^ab^	726 ± 27 ^b^	714 ± 10 ^b^	771 ± 32 ^ab^	837 ± 30 ^a^	693 ± 30 ^b^	785 ± 41 ^ab^	824 ± 19 ^a^	820 ± 46 ^a^	707 ± 19 ^b^
Ʃ Fatty acids	2549 ± 87 ^f^	2730 ± 45 ^e^	2860 ± 72 ^e^	2922 ± 63 ^de^	3205 ± 91 ^bc^	2821 ± 59 ^e^	3195 ± 61 ^bc^	3431 ± 15 ^a^	3313 ± 54 ^ab^	3084 ± 58 ^cd^

Note: The values are expressed as the mean ± standard deviation, based on three parallel experiments; ND: not detected. In the same row, lowercase letters (a–e) are used to indicate results derived from the least significant difference test (*p* < 0.05, Duncan).

**Table 3:** Yeast inoculation modalities.

Inoculation Modalities	*S. cerevisiae*	*W. anomalus*	Inoculation Proportion
SW-4 (5:1)	CECA	Wa-2-84	5:1
SW-5 (5:1)	CECA	Wa-2-85	5:1
SW-8 (5:1)	CECA	Wa-B1-8	5:1
SW-4 (1:1)	CECA	Wa-2-84	1:1
SW-5 (1:1)	CECA	Wa-2-85	1:1
SW-8 (1:1)	CECA	Wa-B1-8	1:1
SW-4 (1:5)	CECA	Wa-2-84	1:5
SW-5 (1:5)	CECA	Wa-2-85	1:5
SW-8 (1:5)	CECA	Wa-B1-8	1:5
Sc	CECA	—	1:0

## Data Availability

All data supporting the conclusions of this article are included in the manuscript.

## Abbreviations

The following abbreviations are used in this manuscript:

ARTP	atmospheric room temperature plasma
OIV	international organization of vine and wine
PCA	principal component analysis
PLSR	partial least squares regression
ARTP	atmospheric room temperature plasma
